# Molecular Diagnosis of Allergy: The Pediatric Perspective

**DOI:** 10.3389/fped.2019.00369

**Published:** 2019-09-24

**Authors:** Stephanie Dramburg, Paolo Maria Matricardi

**Affiliations:** Department of Pediatric Pulmonology, Immunology and Intensive Care Medicine, Charité University Medicine Berlin, Berlin, Germany

**Keywords:** sensitization profile, molecular spreading, allergen-specific IgG, component-resolved diagnostics, prediction

## Abstract

In times of “Precision Medicine” it is fundamental to identify the individual disease phenotype in order to provide an individualized therapy for every patient. This concept is also becoming increasingly important for the treatment of allergic diseases. Thanks to the biological engineering of recombinant and native allergens for the assessment of allergen-specific IgE antibodies, it is now possible to easily obtain the individual sensitization profile of a patient. This allows the allergist to precisely identify the primary elicitor of an IgE response and, based on this knowledge, to choose the best treatment option. Several studies have observed the longitudinal evolution of sensitization profiles and identified a phenomenon termed “molecular spreading,” which describes a broadening of the recognized allergen spectrum from a source over time. Additionally, the identification of marker proteins, which can trigger an IgE response or correlate with an increased risk for certain clinical symptoms, helps to establish an individual risk profile. This information may not only affect the decision-making concerning immunotherapy, but also opens up avenues for future investigations with regard to prevention strategies. We provide here an overview on the role of individual sensitization patterns and their predictive value.

## Molecular Allergology: New Pathways in Allergy Diagnosis

It is long since no secret that allergic diseases are continuously gaining importance in the industrialized world. While many scientists are trying to get to the bottom of these numbers, the situation for a multitude of practitioners is aggravating as they see themselves confronted not only by affected patients demanding treatment, but also with anxious parents seeking recommendations for effective prevention strategies for their children. In order to provide useful information for the doctors to pass on to their patients, research is being performed restlessly, often with changing results. Nevertheless, all research has one least common denominator: in order to prevent a disease and provide proper treatment, it is fundamental to understand the underlying biological and immunological mechanisms from scratch, including those taking place before and finally leading to the onset of the first symptoms.

Since the discovery of IgE antibodies by Ishizaka, Bennich and Johannson in the late sixties, serological tests have become an integral part of the allergological work-up, combined with the clinical history and *in-vivo* methods like skin tests or standardized allergen provocation. While skin tests can also be performed with raw allergens (e.g., prick-to-prick testing), allergenic extracts served as a base for serological assessments during the last decades. As these reagents are sometimes poorly standardized and their composition may vary significantly between preparations from different manufacturers, the precision of results left space for improvement. This gap is now progressively being covered by new diagnostic options based on advanced molecular and structural biology. Thanks to fast-placed technological advances, it became possible not only to identify and purify an increasing number of allergenic molecules with their isoforms, but also to produce them in large quantities through sequencing and cloning. This new opportunity goes along with the need for accurate diagnostic tools in times of “Precision Medicine” which requires the detailed knowledge of the patient's disease phenotype in order to provide individualized treatment options. Molecular singleplex and multiplex assays do not only provide detailed information on the patient's sensitization profile, but also on possible cross-reactivity. They also enable us to observe the longitudinal evolution of complex IgE and IgG repertoires in birth cohort studies, which is fundamental for the perception of pre-clinical immunological phenomena. Once these are understood in depth, more efficient long-term therapies, as well as prophylactic measures could possibly be identified. Of course, humoral responses are only one part of this complex journey, to which the following paragraphs will draw a roadmap.

## The Origins: Allergen-Specific IgG-Responses

**Figure 1:**
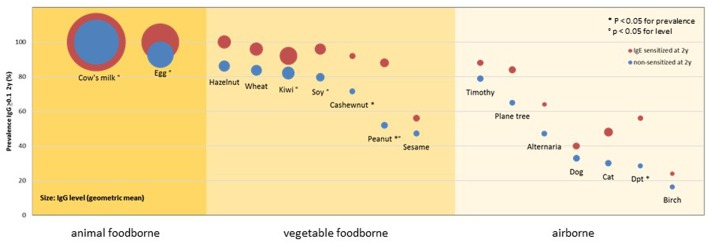
Prevalence and levels of IgG responses to allergenic sources in 148 children of the MAS cohort at 2 years of age by IgE sensitization at 2 years of age and main exposure route. Circles show the prevalence (y-axis) and levels (diameter) of detectable IgG responses (≥0.1 ISU) to allergenic sources in children with (red) or without (blue) IgE sensitization at 2 years of age. *P*-values refer to comparisons of the prevalences and levels [reprinted from (5), Copyright 2016, with permission from Elsevier].

In order to evaluate a child's risk of developing an allergic disease as early as possible, ideally already in a pre-clinical stage, it is fundamentally important to have a clear idea of the immunological processes distinguishing the non-atopic from atopic individuals. As IgE antibodies appear during the first year of life and their quantity is minor, especially when compared to IgG levels, early observations should not be limited to allergen-specific IgE antibodies. The production of allergen-specific IgG has been broadly studied (1), especially in relation to its protective effect as a “blocking antibody” (2) and its role in immunotherapy (3). An analysis of two birth cohorts from Great Britain and Australia showed that IgG, not IgG4, specific to the cat allergen Fel d 1 was able to alter the cat-specific IgE and childhood wheezing association (4). In this study, children with increasing IgG1 levels had a lower risk of developing symptoms. In order to get a broader overview of the early, non-challenged, natural IgG response, we analyzed the sera of 148 atopic as well as non-atopic children toward a broad panel of 91 allergenic molecules from various sources (5). The results obtained at the age of 2 years showed that almost 100% of the children, independently from their atopic status, produced IgG antibodies toward allergenic molecules from foods of animal sources, such as cow's milk and egg. This prevalence, as well as the antibody concentrations, were considerably lower for vegetable foodborne allergens, and lowest for airborne allergens. Parallely, for these two allergen groups, a clear difference in terms of prevalence could be observed among atopic and non-atopic individuals with higher prevalences among the atopic children (Figure 1). Interestingly, antibodies of the IgG4 subclass could be observed only infrequently (<5% of the responses), suggesting that the measured antibodies mainly belong to the IgG1 subclass.

These results indicate that the route and dose of exposure are of prominent importance for the evoked immune response. In order to analyse the effect of different ways of exposure to almost identical allergenic molecules, we examined the natural evolution of allergen-specific IgG antibodies toward the birch-pollen related PR.10 molecules throughout childhood (6). This longitudinal observation showed a clear difference in the IgG responses of birch-atopic patients when compared to non-atopic individuals. Although both groups showed the same type of “default” response in the first 2 years of life, the evolution of this response started to diverge during the third year of life. Non-atopic participants kept the “default” picture of an early and transient response of low concentrations and mainly directed toward foodborne PR-10 molecules; by contrast, those individuals who developed IgE toward the major birch allergen Bet v 1 and its most important homologs, showed a different trend. From the establishment of the IgE response on, a clear increase of specific IgG antibodies toward these molecules could be observed, following an overall hierarchy mostly corresponding to the grade of sequence homology with Bet v 1. This IgG response was not only more prevalent than the earlier observed diffuse pattern, but also showed higher concentrations and a temporal relation to the appearance of IgE antibodies, which could be observed in 93% of the sera simultaneously with or after the beginning of the IgG response. Hence, the contact to minute amounts of birch pollen allergens to the nasal and oral mucosa was able to induce a strong “(pre-)atopic” allergen-specific IgG response in atopic children, while large amounts of regularly consumed PR-10 molecules had no such effect in neither of the study groups.

The chronological interrelation between the IgG and IgE responses further leads to the hypothesis that both isotypes may have been produced by the same B cell subset passing through an indirect class switch. This hypothesis has also been recently supported by immunoglobulin heavy chain mutational lineage data giving evidence that the primary IgE source in human subjects is the secondary isotype switching of mutated IgG1-expressing B cells (7). This fact is crucial as it suggests an ambivalent role of allergen-specific IgG, which has been limited to its protective role and should now be reconsidered as a player in the allergy arena or at least an indicator for a propensity of the immune system toward a Th2-directed, IgE switched response. In addition, Aalberse and Platts-Mills proposed a concept in which chronic allergen exposure with the associated induction of germinal centers prompts IgG4-switched B memory cells. According to the authors, these memory cells may also sporadically undergo a secondary switch to IgE (8).

It has to be mentioned though, that independently from the subclass, the assessment of IgG antibodies has no diagnostic value so far, especially not in food allergy. Further research needs to be performed in order to evaluate its diagnostic and/or predictive value.

## The Initiator: Characteristics of the Early IgE-Response

Once a patient is seeking the opinion of a specialized allergist, he or she is usually already suffering from a significant loss of life quality due to his/her allergic symptoms. The serological assessment of IgE antibodies at this time point then typically shows a strong response toward one or, more likely, already various allergen sources. This information is usually obtained through tests based on allergen extracts. These do provide significant information and are a substantial diagnostic tool, however, especially in poly-sensitized patients, they cannot always reliably indicate the eliciting source primarily responsible for the clinical symptoms (9). The diagnostic strength is dramatically enhanced by the use of allergenic molecules for the detection of specific IgE antibodies (10). By this, cross-reactivity can be revealed and the allergen at fault of the clinical symptoms identified. Of course, the allergist has to keep in mind that the pure presence of IgE antibodies does not indicate an allergy alone. Only the combination with corresponding clinical symptoms makes the diagnosis “allergic disease” possible.

As mentioned before, IgE antibodies are mostly already present in the blood once a patient visits the allergist. But when did this sensitization begin? How long was the pre-clinical phase and what could he observe if the allergist had seen his patient before the onset of symptoms? In order to answer these questions, birth cohorts are an essential pool of resources, which has been used, to assess the longitudinal evolution of IgE responses toward grass pollen molecules in childhood. Their analysis of 177 grass-allergic children revealed a clear structure in the observed sensitization profiles (11). Strikingly, almost all children started their sensitization toward the major grass pollen allergen Phl p 1, some even years prior to the occurrence of clinical symptoms. This phenomenon of an “initiator molecule” starting a sensitization cascade could then also be observed for other allergen sources. For example, children and adolescents allergic to birch pollen showed a primary sensitization to Bet v 1 in various independent studies from Germany (6), Austria (12), and Sweden (13). A sera analysis of 722 participants of the German Multicenter Allergy Study (MAS) for sensitization patterns toward house dust mite revealed that the major allergens Der p 1, Der p 2, and Der p 23 are the proteins most frequently initiating an IgE response (14). The knowledge about the existence of these initiator molecules leads to the question about their exact biological function and influence on a future spreading of the sensitization pattern. Once these questions will be answered, initiator molecules may form a strategic point of action for screening and secondary prevention.

## Individual Sensitization Patterns and the Phenomenon of “Molecular Spreading”

**Figure 2:**
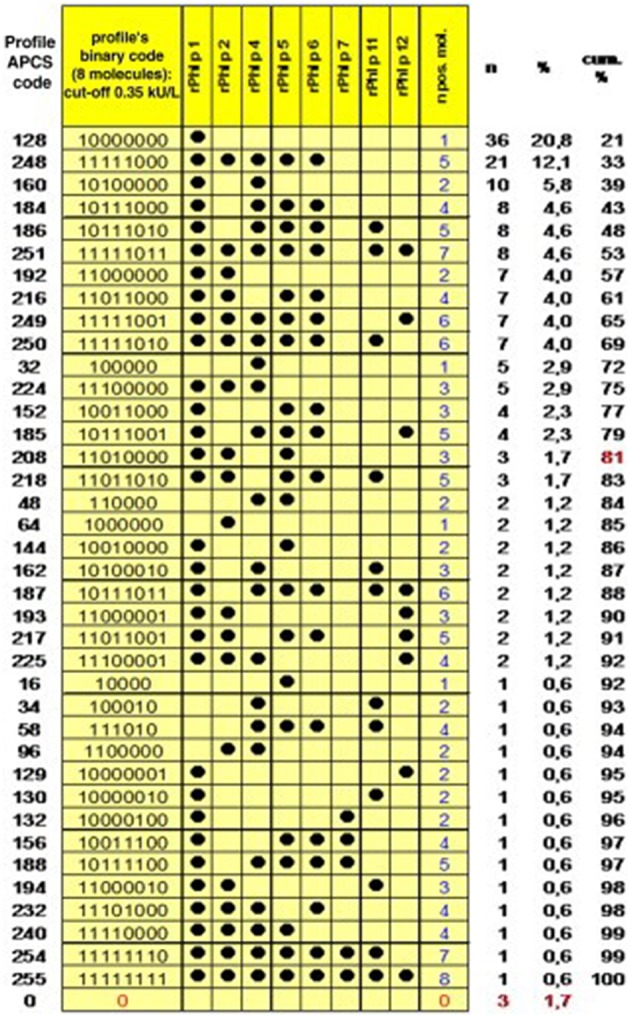
Profiles of IgE sensitization to 8 P pratense molecules in 176 children with an IgE reaction to P pratense and complete dataset. The Allergen Profile Codification System (APCS) code and the absolute and cumulative frequencies are shown. The profiles are ordered by decreasing frequency, and the point at which the arbitrary threshold of 80% of the patient population has been reached is marked in red [reprinted from (15), Copyright 2012, with permission from Elsevier].

After the identification of initiator molecules, the subsequent course of sensitization is of fundamental interest. An analysis of the individual IgE profiles among 176 grass-pollen-allergic children in Italy resulted in the identification of 39 different sensitization patterns (Figure 2). This broad heterogeneity shows that every immune system reacts individually to a stimulus, which in this case is assumed equal for all participants, as they have been entirely recruited within the city Rome (15). Interestingly, when matching the obtained sensitization profiles with a molecularly designed preparation for specific immunotherapy (SIT) as a component-resolved treatment (CRT), only 4% of the profiles matched the balanced and standardized mixture of Phl p 1, Phl p 2, Phl p 5a and Phl p 5b, and Phl p 6. These results suggest a wide heterogeneity of sensitization profiles toward *Phleum pratense* molecules. Nevertheless, Hatzler et al. were able to identify a clear progression in the IgE patterns of 177 participants of the MAS study. While in over 75% of the cases the IgE response was clearly initiated by Phl p 1, further responses mainly appeared according to the following patterns: Phl p 1, then Phl p 4 and Phl p 5; then Phl p 2, Phl p 6, and Phl p 11; and then Phl p 12 and Phl p 7.

**Figure 3:**
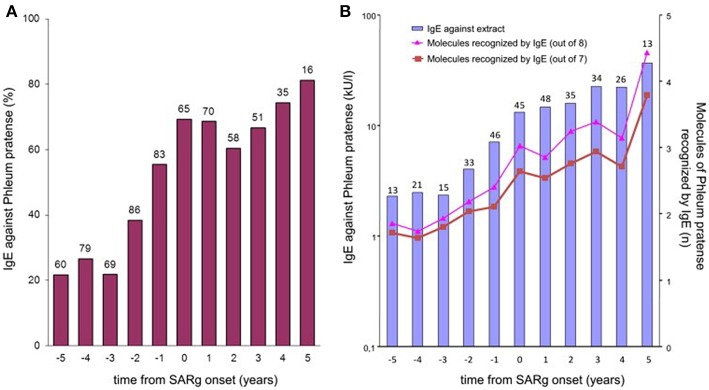
IgE to P pratense by time from onset of grass-related seasonal allergic rhinitis. **(A)** Bars show the prevalence of IgE sensitization (≥0.35 kUA/L) to P pratense (extract) in children whose sera were available at each point in time. The number of tested children is indicated over each bar. **(B)** Bars show the geometric mean levels of IgE antibodies to P pratense (extract) at each point in time in IgE antibody–positive sera. The numbers of tested children is indicated over each bar. Lines show the average number of all 8 (triangles) or only 7 (squares; Phl p 6 excluded) allergenic molecules of P pratense recognized by IgE antibodies at each point in time in IgE antibody–positive sera [reprinted from (11), Copyright 2012, with permission from Elsevier].

Strikingly, many subjects had produced IgE toward various allergenic grass pollen molecules already up to several years before the onset of disease. An analysis of IgE profiles before and after the beginning of clinical symptoms additionally showed that not only the complexity, but also the levels of the IgE responses are significantly lower before the first occurrence of symptoms (Figure 3). This sequential evolution of an immune (IgE-) response toward different, non-cross-reactive molecules of the same antigen/allergen source has been termed “molecular spreading” underlining the importance of an initiator molecule as the trigger of this phenomenon. A recent study among 59 grass pollen allergic children from Greece further suggested the usefulness of component-resolved diagnosis (CRD) as an indicator for disease severity as the presence of IgE antibodies to the *Phleum pratense* molecules Phl p 1, Phl p 2, Phl p 5, and Phl p 6 were associated significantly with moderate to severe symptoms (16).

**Figure 4:**
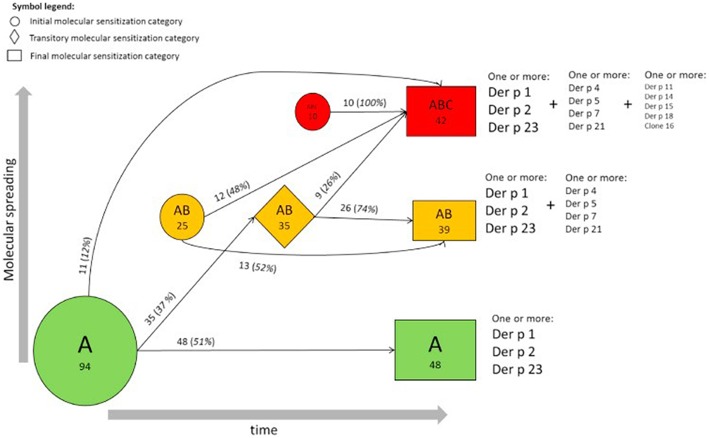
Trajectories of IgE sensitization in mite-sensitized subjects (*n* = 129). Evolution of the IgE responses to 12 D pteronyssinus allergen molecules according to the A, AB, or ABC classification in participants sensitized at 2 or more follow-up points is shown. The round, rhombus, and rectangular boxes represent the initial, intermediate, and final sensitization stages, respectively. Numbers (percentages) refer to participants, and areas are proportional to their frequency [reprinted from (14), Copyright 2017, with permission from Elsevier].

Focusing on the concept of a “molecular spreading” in house dust mite allergy, the sera of 722 subjects from the MAS cohort were analyzed for IgE to 12 recombinant house dust mite (*Dermatophagoides pteronyssinus*) allergens (14, 17). The obtained results provided a comprehensive view of the longitudinal evolution of mite-specific IgE responses from birth up to the age of 20 years. As mentioned before, the observed responses were most frequently triggered by the “initiator molecules” Der p 1, Der p 2, and Der p 23 (a recently identified major allergen). Interestingly, an inverse correlation was observed between the mean age at first detection and the frequency of detection of each molecule with the strongest initiators of an IgE response being the ones inducing the earliest response. To discern the myriad of IgE responses observed, we proposed a stratification system according to a prevalence ranking at 20 years of age. This strategy assorted the 12 molecules into 3 groups, according to the frequency of evoked IgE responses. The molecules with the highest frequency (Der p 2, Der p 1, Der p 23) were assigned to group A, the secondary molecules to group B (Der p 5, Der p 4, Der p 7, Der p 21), and the less relevant molecules to group C (Der p 11, Der p 14, Der p 15, Der p 18, and Clone 16). Regarding the patients' sensitization patterns, it was observed that IgE was most frequently directed against the molecules belonging to group A, progressing then to group B, and later to group C (Figure 4). However, it is important to mention that not all children followed the entire trajectory, but individual sensitization profiles were rather heterogeneous at age 20. At this timepoint, 27 children responded to only one molecule (monomolecular profile), 50 to 2–4 (oligomolecular profile), and 42 to more than 5 of the 12 molecules (polymolecular profile). A recent study among 276 Spanish children confirmed the importance of the allergenic mite molecules previously identified as trigger with 86.6% of the patients being sensitized to nDer p 1, 79.3% to rDer p 2 and 75.8% to rDer p 23. Notably, 26 participants were not sensitized to Der p 1 and Der p 2 and 14 of these had positive IgE binding only to Der p 23. Children suffering from allergic asthma, especially those with a persistent moderate and severe phenotype were more often sensitized to all the three major allergens (Der p 1, Der p2, Der p 23) (18).

Earlier, Custovic et al. tested the sera of 235 children of the Manchester Asthma and Allergy Study birth cohort for specific IgE toward grass pollen and mite allergens (19). This study revealed three differential classifications with regard to the first observation of IgE toward grass pollen: (1) no/low sensitization; (2) early onset; and (3) late onset. While the early-onset trajectory was associated with asthma and diminished lung function, children of the late-onset trajectory suffered more frequently from rhinitis. Further, three distinctive sensitization trajectories linked to mite allergy occurring between 5 and 11 years were identified: group 1 to Der f 1 and Der p 1, group 2 to Der f 2 and Der p 2, and “complete” mite sensitization (to both groups, and in addition to Der p 10, Blo t 5 from *Blomia tropicalis*, and Lep d 2 from *Lepidoglyphus destructor*). Children belonging to group 1 and to the “complete” mite sensitization group presented a significantly increased risk for asthma, eczema and rhinitis, although the “complete” mite sensitization group had the highest odds ratio for asthma. Unlike the children with sIgE to grass pollen, the longitudinal IgE-evolution had no impact on mite-allergic subjects. In summary, individual sensitization patterns toward airborne allergens can be described as diverse but an overall trend toward a broadening of the IgE response with clinical relevance over time is common to most patients.

## Predictive Value of Specific IgE-responses to Allergenic Molecules

The presence of allergen-specific IgE antibodies toward airborne allergens has been lately verified as a predictive marker for the development of asthma throughout childhood and adolescence (20). In the case of seasonal allergic rhinitis to grass pollen, a similar predictive potential had been observed previously, which was significantly enhanced by involving the parental history of hay fever (21). Component-resolved diagnosis (CRD) allows to precisely identify the number and type of recognized molecules in the individual patient as well as his or her IgE responsiveness toward certain marker molecules. By this, individual risk profiles can be established based on the knowledge of certain marker proteins, such as the grass pollen allergen Phl p 12 which goes along with an increased risk for the development of an Oral Allergy Syndrome (OAS). Also other *Phleum pratense* proteins are related to higher probabilities of certain clinical outcomes, such as e.g., Phl p 7 which has been shown to be associated with a higher risk of developing asthma (10). On the other hand, low levels of Ige toward Phl p 5 have been related to a low prevalence of asthma among 140 Italian patients (22). In general, several studies have proven that a polysensitization toward a broad panel of allergenic molecules from one source increases the risk for and severity of clinical symptoms upon allergen contact.

Going back to the above mentioned stratification system of sensitization patterns in house dust mite allergy, we showed that subjects within the broadest sensitization stage (i.e., A->B->C) had a significantly elevated risk of mite-related allergic rhinitis and asthma, or both, than not sensitized participants. The authors could identify various factors leading to a broadening of the IgE repertoire. Especially an early onset of sensitization, a history of parental hay fever, and higher levels of exposure to mites could be identified as parameters associated with a more complex, polymolecular IgE sensitization pattern. Furthermore, this study also discovered certain marker molecules, as IgE to Der p 1 and/or Der p 23 at the age of 5 or younger predicted a higher risk of developing asthma at school age (i.e., 6–20 years).

In birch pollen allergy, increased risk of incidence and persistence of seasonal allergic rhinitis up to the age of 16 years was shown to correlate with increasing levels of Bet v 1-specific IgE or increasing numbers of IgE-reactive PR-10 proteins at the age of 4 years. This observation has been made among 764 children from the Barn/Children Allergi/Allergy Milieu Stockholm Epidemiologic (BAMSE) study birth cohort who were examined at the age of 4, 8, and 16 years. Additionally to these predictive results, some general observations on the evolution of IgE responses toward birch-related allergens were made. Confirming our previous observations (6), the prevalence of IgE sensitization increased over time, reaching in this study 25% at age 16 years. Furthermore, the IgE reactivity of PR.10 proteins displayed the following hierarchic relationship: Bet v 1 > Mal d 1 > Cor a 1.04 > Ara h 8 > Pru p 1 > Aln g 1 > Api g 1 > Act d 8 > Gly m 4.

An analysis of the sera of 779 children selected from the same cohort has shown that IgE to the major cat and dog allergen molecules Fel d 1 (cat) and Can f 1 (dog), allows a significantly better prediction of cat and dog allergy (cross-sectionally and longitudinally) than IgE assessed with cat and dog extract. The same could be observed for a polysensitization to either cat or dog allergen molecules, underlining the advantage of CRD in allergy diagnostics.

## Implications for Immunotherapy

The expanded information obtained through molecular allergy diagnostics raises new questions, not only concerning the decision for or against an immunotherapy, but also on the correct timing for its application as well as the composition of therapeutic agents (23). Although an allergen-specific immunotherapy is still the only disease-modifying treatment, many patients are only treated with symptom-relieving drugs, especially when the eliciting allergen source cannot be precisely identified by the assessment of clinical history and skin prick testing. An Italian study following 651 pollen-allergic children in 16 different outpatient clinics showed that the prescription behavior of the attending allergists changed significantly, when providing molecule-based serological results of their patients. Depending on the guidelines (European, American, own experience), the decision in favor of an immunotherapy treatment changed for 42–48% of the patients (24). This emphasizes the value of molecular allergy diagnostics for clinical decision-making. However, the assumed improvement of SIT efficacy when based on CRD-guided prescription yet has to be investigated in prospective studies.

An important factor influencing the efficacy of any therapy is its suitability for the disease to be treated. As described previously, our theoretical exercise showed that in only 4% of the 176 examined grass-pollen allergic patients, the sensitization profile matched with the composition of a standardized immunotherapy solution (15). In 29% of the remaining 169 subjects, the therapeutic agent would have been “underpowered,” whereas 32% were clearly over-treated with molecules they had not produced IgE antibodies against yet. While the efficacy of an underpowered treatment is most likely to be low, the risk of inducing new sensitizations by including unnecessary molecules in the therapeutic agent could be considered. Although not yet commercially available, a tailored immunotherapy, including only the molecules of the patient's individual sensitization profile, might be the most promising in terms of treatment efficiency and safety (25).

Another option to increase the success rates of immunotherapy is a good timing. Unfortunately, most of the treatments are currently being initiated only years after the first onset of symptoms and often after a significant loss of life quality. Many patients and attending doctors first observe the natural course of clinical symptoms and especially the former hope for their symptoms to dissolve, without potentially painful treatment with subcutaneous injections. Unfortunately only very few of the clinical manifest allergies tend to dissolve naturally. On the contrary, many mono-sensitized children—especially those with parents affected by hay fever (21)—tend to broaden their sensitization profile consecutively even toward molecules from additional allergen sources, e.g., in birch allergic patients developing a grass-pollen allergy. Although there's no clear recommendation for the best time point to start an immunotherapy, studies on molecular spreading suggest that “the earlier, the better (25).”

## Future Perspectives—Immunoprophylaxis

**Figure 5:**
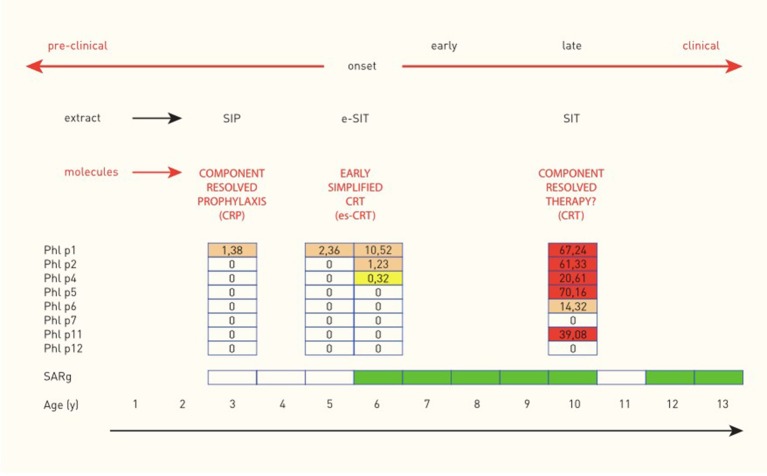
Molecular spreading of the IgE response to Timothy grass and potential implications for allergen-specific immunologic intervention in a child with seasonal allergic rhinitis to grass pollen (SARg). Molecular spreading of the IgE response to Phleum pratense and implications for allergen-specific immunological intervention in one child with hay fever (case from the MAS birth cohort) [reprinted with permission from (23), https://journals.lww.com/co-allergy/pages/default.aspx].

As mentioned previously in this chapter, a “molecular spreading” phenomenon has been described for various airborne allergens with the same final outcome: patients with a broad spectrum of IgE to many molecules of one source are more likely to suffer from more severe clinical symptoms than those exhibiting a less complex sensitization profile. Reasoning about potential starting points to prevent this late clinical phase ends in two to three options for treatment (Figure 5). Currently, the most common option is to start an allergen-specific immunotherapy once symptoms have aggravated and cannot be properly controlled by symptom-relieving drugs (antihistamines, corticosteroids) anymore. The therapeutic agents used for this treatment usually consist of allergen extracts or standardized mixtures of molecules including the most important major allergens. Unfortunately, this treatment strategy bears the risk of over- or under-treatment and an individually tailored therapy may be more effective but is not yet established. Another approach is preventing the aggravation of symptoms by intervening at an early clinical stage, right after the onset of symptoms. At this stage, the molecular spreading process has not yet fully evolved and sensitization profiles are mostly simple. Consequently, such a customized SIT containing exclusively the targeted molecules might prevent a further spreading of the IgE response. Taking this theoretic reasoning even one step further, even the pre-clinical phase may be of interest for an early intervention. It has been shown that healthy pre-school children with IgE to grass pollen are likely to develop a seasonal allergic rhinitis triggered by grass pollen at school age, especially when their parents suffer from hay fever (11, 21). Children fulfilling these two criteria may therefore be a target for prevention via pre-clinical allergen-specific immunoprophylaxis (26). The target of this intervention would be to prevent the onset of seasonal allergic rhinoconjunctivitis symptoms, by keeping the IgE response to grass pollen at a subclinical level.

A recent meta-analysis of 32 studies, however, could not depict a statistically significant reduction of the risk to develop a first allergic disease by early allergen-specific immunotherapy (AIT) (27). Yet, a diminution of the short-term risk for asthma development in allergic rhinitis patients who received AIT could be demonstrated. Whether early AIT also has an effect on the development of asthma in a long-term prospect is still unclear though.

This means that, albeit the disease prevention is not possible, its onset could be delayed and its symptoms moderated, maybe even prevented with regard to asthma.

## Author Contributions

Both authors have made a substantial, direct and intellectual contribution to the work, and approved it for publication.

### Conflict of Interest Statement

The authors declare that the research was conducted in the absence of any commercial or financial relationships that could be construed as a potential conflict of interest.
